# The Probable Manner of Attachment of Implanted Teeth

**Published:** 1888-09

**Authors:** 


### THE PROBABLE MANNER OF ATTACHMENT OF IMPLANTED TEETH.

The dental profession has become familiar with the operation of implantation as devised by Dr. Younger, of San Francisco, by clinics given by him and others in nearly all our prominent dental societies. The mechanical part of the operation has been fully demonstrated, and is thoroughly successful. The scientific and therapeutic part, however, and the special details that tend toward final success or failure are not so well known. Sufficient time has not elapsed since the operation was first introduced to allow of a final verdict in the case; enough, however, has been done to disprove the first assertion that the operation was unsurgical. It is true that Dr. Younger's ideas regarding the method of attachment were crude, but he had the practical experience behind him that could not be gainsaid, and which secured for him a respectful hearing from the profession. The theory of the revivification of the pericementum was so utterly absurd that, had it not been for the practical demonstration of the fact that teeth so implanted could and did become firmly set in the jaw, it would have been sufficient to have overthrown the entire operation.

An operation that now promises to be of real benefit to humanity was introduced and held its own through merit, although based upon such an unscientific hypothesis that upon its first presentation it received the appellations of absurd and unsurgical, and the leading oral clinician in the country refused its demonstration before his students. In this age of high scientific attainments, only those operations are entertained that are based upon scientific principles. The scientific aspect is first studied out, and the operation results. The opposite was, however, the case in implantation. The method of attachment was an enigma, and different theories regarding the nature of the attachment were advanced. We were called upon early in the history of the operation for our opinion regarding it. The cases that had come under our notice were limited, and we had had no opportunity to make any experiments or histological examinations of teeth that had been implanted. Knowing, however, the nature of organization of exudated lymph, when such does occur, we said that the attachment was probably fibrous, and did not depend upon the revivification of the pericementum. Time has proven the correctness of this statement, and that such is the first stage in the method of attachment. The plasma cells that are thrown out become organized, and a firm connective tissue envelops and encircles the root. The fine processes of the cells probably penetrate the superficial lacunæ of the cementum, and in this way serve to help attach the tooth more firmly than would otherwise be the case were a porcelain tooth used. Such may be said to be the manner of attachment at the end of the first week or ten days after operation; but a second stage intervenes, provided the inflammation is kept in abeyance, when the process of ossification sets in and proceeds until the socket is filled with new formed bone, giving rise to a bony ankylosis. No experiments have been made to demonstrate the truth of this latter statement; but the solidity with which the teeth become attached, the lack of mobility and the peculiar resonant sound given off when such teeth are tapped, all tend to confirm our opinion regarding the nature of the attachment in successful cases. We have not been able to induce any person who has had a tooth successfully implanted to sacrifice himself for the cause of science, and allow the extraction of the tooth with a portion of the alveolar wall. The cases that have been examined were where the teeth had been lost or where the operation had not been an entire successs, and were, consequently, not suitable cases for investigation. We are promising ourself this winter to make some experiments in this direction if the time can possibly be found to do so.

THE PROGNOSIS.

We look very favorably upon the operation, and consider it far from unsurgical as at present practiced by those of our acquaintances. To Dr. E. C. Kirk, perhaps, belongs the credit of doing most towards putting the operation upon a thoroughly scientific basis. He has given the subject a great deal of attention, and the operation as practiced by him seems to meet all the requirements. The success of the operation depends upon three things; first, the selection of the right kind of a case upon which to operate; second, antiseptically performing the operation; and third, the maintenance of the proper equilibrium between progressive and retrogressive forces after the operation. Under the first head there can be no question but that persons in whom there is a scrofulous or even a catarrhal tendency will make unfavorable cases upon which to operate. The first result after operation is exudation of lymph, following which one of two things occurs, the lymph either organizes or breaks down. The latter is most apt to result in individuals suffering from a scrofulous or catarrhal diathesis. The same holds good for those individuals in either the secondary or tertiary stages of syphilis. So long as there is any tendency toward retrograde metamorphosis, there can be no assurance of success. The secret lies in keeping such changes in abeyance, and stimulating the up-building process, which tends to organization. We think that the process of implantation gives much more promise of success than does re- or transplantation. The fact that in implantation we have perfectly healthy bone surrounding the root implanted is a point very decidedly in favor of a quick and favorable healing of the tissues by first intention. In re- or transplantation, an old and, in most cases, diseased socket is utilized, and if good results are obtained it is first necessary to overcome the retrogressive tendency already situated in the tissues; whereas in implantation, in favorable cases, nature is on our side. Then, again, the operation of implantation comes forward at a time when we are much more conversant with the physiological and pathological laws that control inflammation, and as that is one of the essential features, a knowledge of which conduces to the success of the operation, we therefore look for good and sufficiently permanent results to entitle it to a place among accepted surgical operations.

### A WORD OF CAUTION.

No one can but help noticing the fact that the dentist of to-day is called upon to do more difficult tasks than formerly, and also to assume more responsibility. This is notably the case in implantation, and it may not be amiss to call attention to the legal responsibility a dentist assumes in performing surgical operations, the instruction in the methods of which is not generally embodied in the dental curriculum, and regarding which a doubt exists as to whether the possession of the dental degree will guarantee protection in case of legal prosecution for malpractice. There can be no doubt but that very serious results may arise from implantation of teeth, such as the transmission of syphilis, pyemia and tetanus. We very much doubt whether a jury of the present day would exonerate an operator practicing on his D. D. S. if such result should occur, especially if he could not demonstrate his entire fitness to perform such operation and treat all subsequent symptoms that might arise. It is generally accepted that thorough antiseptic precautions will reduce the danger of infection almost if not entirely, but he who would assume the responsibility ought surely to thoroughly inform himself upon these points, and be able to defend himself before a jury. Thus are the necessities for broader education being constantly brought to the notice of the profession. Dentistry cannot any longer be practiced as a trade, but he who would live up to all the grand possibilities of our grand profession must know the scientific as well as the mechanical part of dentistry. We make reference to these facts not to discourage any from practicing implantation, but to show the necessity for becoming thoroughly conversant with the scientific as well as the mechanical phase of the operation in order to be assured of all the protection possible in case of bad results which are apt to fall to any who perform surgical operations.

